# Supplementary material for: Toxicogenomics Data for Chemical Safety Assessment and Development of New Approach Methodologies: An Adverse Outcome Pathway‐Based Approach
Source: Adv Sci (Weinh). 2022 Dec 7;10(2):2203984. doi: 10.1002/advs.202203984 (PMC9839874; doi:10.1002/advs.202203984)
Supplement: Supplementary file 1 — Supporting Information [file ADVS-10-2203984-s001.pdf]

ADVANCED SCIENCE
Open Access

Supporting Information

for *Adv. Sci.*, DOI 10.1002/advs.202203984

Toxicogenomics Data for Chemical Safety Assessment and Development of New Approach Methodologies: An Adverse Outcome Pathway-Based Approach

*Laura Aliisa Saarimäki, Jack Morikka, Alisa Pavel, Seela Korpilähde, Giusy del Giudice, Antonio Federico, Michele Fratello, Angela Serra and Dario Greco**

Supporting Information

Toxicogenomics Data for Chemical Safety Assessment and Development of New Approach Methodologies: an Adverse Outcome Pathway -based Approach

*Laura Aliisa Saarimäki, Jack Morikka, Alisa Pavel, Seela Korpilähde, Giusy del Giudice, Antonio Federico, Michele Fratello, Angela Serra, and Dario Greco**

*Corresponding author: dario.greco@tuni.fi

1. Supporting Materials and Methods

1.1. Data Integration into the Unified Knowledge Space

The previously introduced Knowledge Graph framework, the Unified Knowledge Space (UKS) was expanded with AOP data as well as additional sources of protein-protein interaction (PPI) and transcription factor-gene associations. The UKS is managed in Neo4j v. 4 (https:// https://neo4j.com/). A full list of data sources used in this study are presented in **Table S1**.

Adverse Outcome Pathways and Key Events were downloaded from AOP-Wiki via their json files. Relationships between Key Events were downloaded from AOP-Wiki as a .tsv file (aop_ke_ker3.tsv). Data included in the annotation framework was downloaded in November 2020 and updated in October 2021. Each AOP was assigned its own node entity and its associated Key Events were linked to it. Since the same KE can be mapped to multiple AOPs and contain distinct Key Event Relationships (connections to other Key Events), an AOP specific Key Event entity type was created in addition to the parent Key Event entities. This allows to capture the individual relationships between KEs for a specific AOP, while through their hierarchical relationship, shared KEs/AOPs are still easily obtained. This data schema is outlined in **Figure S1**. Known stressors for an AOP were also linked to their corresponding AOP node(s) and labels denoting properties such as molecular initiating event and adverse outcome were added as additional node labels to their corresponding (specific) key event nodes based on the data retrieved from AOP-Wiki.

All the individual PPI and gene regulation data sets were mapped to their corresponding Ensembl Gene IDs, which represent GENE nodes in the UKS (**Figure S1**). An edge was added between two nodes, if it is present in at least one of the downloaded data sets. However, to ensure transparency for each edge, all sources supporting it were added as an edge attribute. These attributes can later serve as a reliability score to create a robust gene–gene network (applies to the PPI data only) (1). Similarly, the collected gene sets (i.e., Pathways, GO, Phenotypes) were added to the UKS as individual node types. They were linked to their associated genes (**Figure S1**), and again, for each edge its supporting sources were retained. Gene sets that may be similar but do not come from the same system were not merged, since while they may be highly similar, there is no official one-to-one mapping between gene sets. Hierarchical information between nodes, such as that present in GO and HPO were also added to the UKS. Due to technical reasons previously explained in Pavel, del Giudice et al. (1), all genes and their gene products have been mapped to Ensembl Gene IDs, and hence will be referred to as GENE.

Table S1: List of resources integrated into the UKS knowledge graph.

Data type	Resource	Reference
AOPs	Aop-Wiki	https://aopwiki.org/aops.json
KEs	Aop-Wiki	https://aopwiki.org/events.json
KERs	Aop-Wiki	https://aopwiki.org
Genes and gene products	Ensembl	(2)
Pathways	KEGG	(3)
	WikiPathways	(4)
	Reactome	(5)
Phenotypes	Human Phenotype Ontology	(6)
	KEGG disease	(3)
Gene ontologies	Gene Ontology	(7)
Chemical-gene associations	CTD	(8)
Protein-protein interaction	HIPPIE	(9)
	HitPredict	(10,11)
	HuRi	(12)
	HI-union	(12)
	Lit-BM	(12)
	Yang-16	(13)
	HI-II-14	(14)
	PINA	(15)
	MINT	(16)
	InnateDB	(17)
	KEGG	(3)
	Reactome	(5)
	PhosphoNetworks	(18)
	SignaLink	(19)
	STRING	(20)
	Pharos	(21)
Transcription factor - gene interaction	TRRUST	(22)
	TransmirR	(23,24)
	JASPAR	(25)
	miRTarBase	(26)

Supplementary References

1. Pavel A, del Giudice G, Federico A, Di Lieto A, Kinaret PAS, Serra A, et al. Integrated network analysis reveals new genes suggesting COVID-19 chronic effects and treatment. Brief Bioinformatics. 2021;

2. Cunningham F, Allen JE, Allen J, Alvarez-Jarreta J, Amode MR, Armean IM, et al. Ensembl 2022. Nucleic Acids Res. 2022 Jan 7;50(D1):D988–95.

3. Kanehisa M, Goto S. KEGG: Kyoto encyclopedia of genes and genomes. Nucleic Acids Res. 2000 Jan 1;28(1):27–30.

4. Martens M, Ammar A, Riutta A, Waagmeester A, Slenter DN, Hanspers K, et al. WikiPathways: connecting communities. Nucleic Acids Res. 2021 Jan 8;49(D1):D613–21.

5. Gillespie M, Jassal B, Stephan R, Milacic M, Rothfels K, Senff-Ribeiro A, et al. The reactome pathway knowledgebase 2022. Nucleic Acids Res. 2022 Jan 7;50(D1):D687–92.

6. Köhler S, Gargano M, Matentzoglu N, Carmody LC, Lewis-Smith D, Vasilevsky NA, et al. The human phenotype ontology in 2021. Nucleic Acids Res. 2021 Jan 8;49(D1):D1207–17.

7. Ashburner M, Ball CA, Blake JA, Botstein D, Butler H, Cherry JM, et al. Gene Ontology: tool for the unification of biology. Nat Genet. 2000 May;25(1):25–9.

8. Davis AP, Grondin CJ, Johnson RJ, Sciaky D, Wiegers J, Wiegers TC, et al. Comparative Toxicogenomics Database (CTD): update 2021. Nucleic Acids Res. 2021 Jan 8;49(D1):D1138–43.

9. Alanis-Lobato G, Andrade-Navarro MA, Schaefer MH. HIPPIE v2.0: enhancing meaningfulness and reliability of protein-protein interaction networks. Nucleic Acids Res. 2017 Jan 4;45(D1):D408–14.

10. Patil A, Nakai K, Nakamura H. HitPredict: a database of quality assessed protein-protein interactions in nine species. Nucleic Acids Res. 2011 Jan;39(Database issue):D744-9.

11. López Y, Nakai K, Patil A. HitPredict version 4: comprehensive reliability scoring of physical protein-protein interactions from more than 100 species. Database (Oxford). 2015 Dec 26;2015.

12. Luck K, Kim D-K, Lambourne L, Spirohn K, Begg BE, Bian W, et al. A reference map of the human binary protein interactome. Nature. 2020 Apr 8;580(7803):402–8.

13. Yang X, Coulombe-Huntington J, Kang S, Sheynkman GM, Hao T, Richardson A, et al. Widespread expansion of protein interaction capabilities by alternative splicing. Cell. 2016 Feb 11;164(4):805–17.

14. Rolland T, Taşan M, Charloteaux B, Pevzner SJ, Zhong Q, Sahni N, et al. A proteome-scale map of the human interactome network. Cell. 2014 Nov 20;159(5):1212–26.

15. Du Y, Cai M, Xing X, Ji J, Yang E, Wu J. PINA 3.0: mining cancer interactome. Nucleic Acids Res. 2021 Jan 8;49(D1):D1351–7.

16. Licata L, Briganti L, Peluso D, Perfetto L, Iannuccelli M, Galeota E, et al. MINT, the molecular interaction database: 2012 update. Nucleic Acids Res. 2012 Jan;40(Database issue):D857-61.

17. Breuer K, Foroushani AK, Laird MR, Chen C, Sribnaia A, Lo R, et al. InnateDB: systems biology of innate immunity and beyond--recent updates and continuing curation. Nucleic Acids Res. 2013 Jan;41(Database issue):D1228-33.

18. Hu J, Rho H-S, Newman RH, Zhang J, Zhu H, Qian J. PhosphoNetworks: a database for human phosphorylation networks. Bioinformatics. 2014 Jan 1;30(1):141–2.

19. Csabai L, Fazekas D, Kadlecsik T, Szalay-Bekő M, Bohár B, Madgwick M, et al. SignaLink3: a multi-layered resource to uncover tissue-specific signaling networks. Nucleic Acids Res. 2022 Jan 7;50(D1):D701–9.

20. Szklarczyk D, Gable AL, Lyon D, Junge A, Wyder S, Huerta-Cepas J, et al. STRING v11: protein-protein association networks with increased coverage, supporting functional discovery in genome-wide experimental datasets. Nucleic Acids Res. 2019 Jan 8;47(D1):D607–13.

21. Sheils TK, Mathias SL, Kelleher KJ, Siramshetty VB, Nguyen D-T, Bologa CG, et al. TCRD and Pharos 2021: mining the human proteome for disease biology. Nucleic Acids Res. 2021 Jan 8;49(D1):D1334–46.

22. Han H, Cho J-W, Lee S, Yun A, Kim H, Bae D, et al. TRRUST v2: an expanded reference database of human and mouse transcriptional regulatory interactions. Nucleic Acids Res. 2018 Jan 4;46(D1):D380–6.

23. Wang J, Lu M, Qiu C, Cui Q. TransmiR: a transcription factor-microRNA regulation database. Nucleic Acids Res. 2010 Jan;38(Database issue):D119-22.

24. Tong Z, Cui Q, Wang J, Zhou Y. TransmiR v2.0: an updated transcription factor-microRNA regulation database. Nucleic Acids Res. 2019 Jan 8;47(D1):D253–8.

25. Castro-Mondragon JA, Riudavets-Puig R, Rauluseviciute I, Lemma RB, Turchi L, Blanc-Mathieu R, et al. JASPAR 2022: the 9th release of the open-access database of transcription factor binding profiles. Nucleic Acids Res. 2022 Jan 7;50(D1):D165–73.

26. Huang H-Y, Lin Y-C-D, Li J, Huang K-Y, Shrestha S, Hong H-C, et al. miRTarBase 2020: updates to the experimentally validated microRNA-target interaction database. Nucleic Acids Res. 2020 Jan 8;48(D1):D148–54.

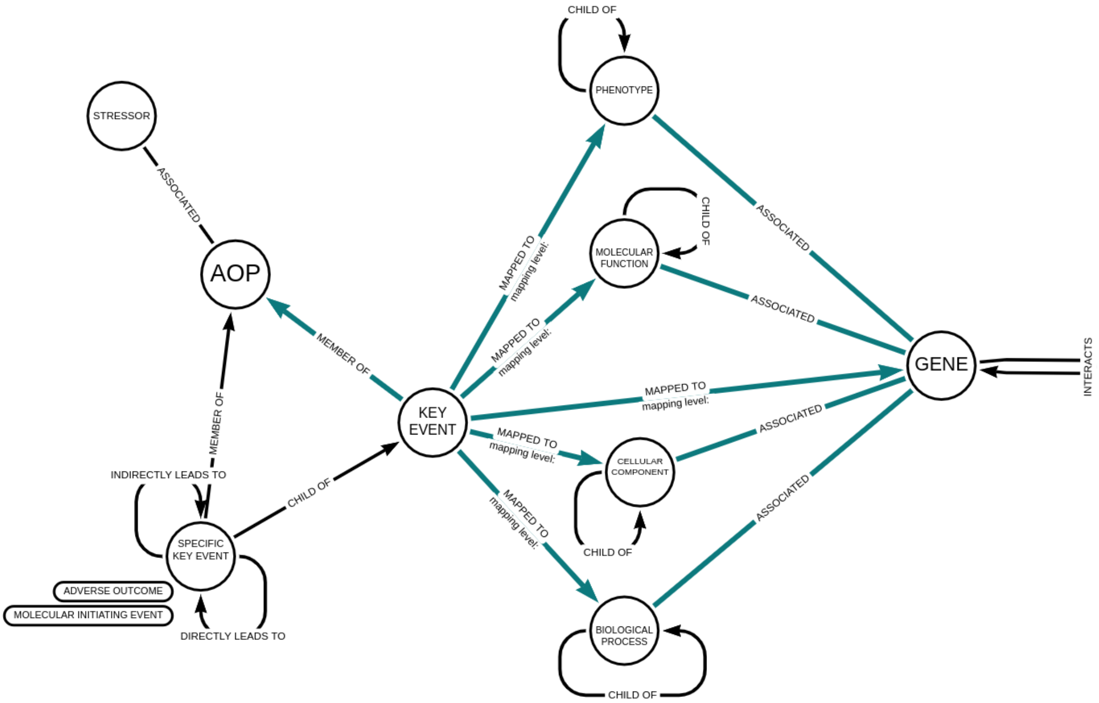

Figure S1. Data schema of the Unified Knowledge Space (UKS). Circles represent nodes with examples of different node types included. Arrows denote directed edges while lines without arrowhead correspond to undirected connections. Examples of relationships are provided as a label to edges. Blue edges describe the connections introduced into the UKS for AOP data.

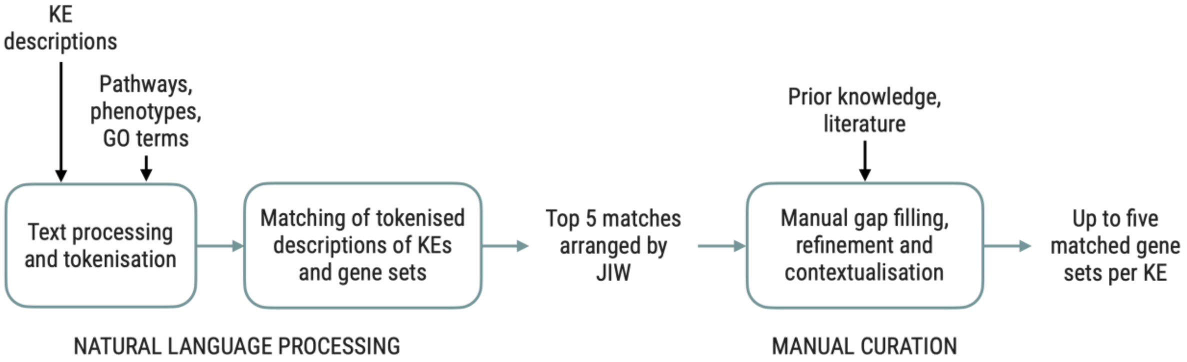

Figure S2. Key event (KE) annotation pipeline comprising natural language processing technigues and manual refinement. Outline boxes describe the steps of the pipeline while inputs and outputs are denoted as text and arrows. JIW = Weighted Jaccard Index.

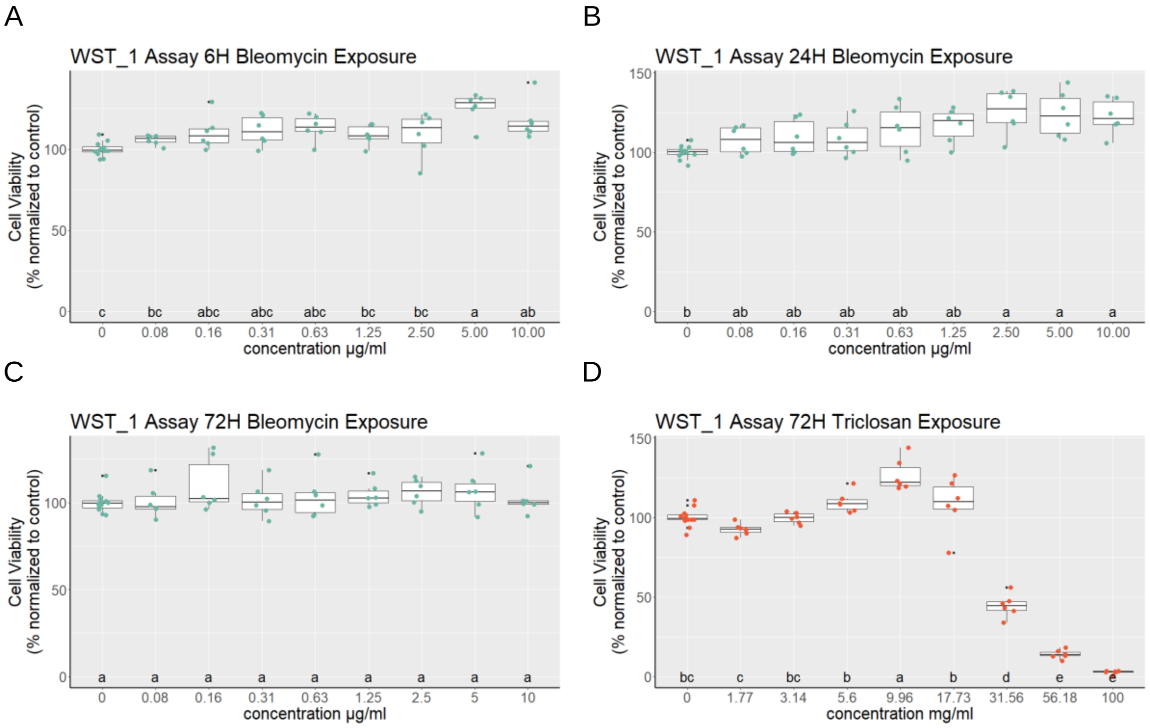

Figure S3. Viability of THP-1 cells exposed to 0-10µg/ml of bleomycin and 0-100µg/µl of triclosan (control). Green points represent individual measures of cells exposed to bleomycin with n = 12 at concentration 0µg/ml and n = 6 for all other concentrations. Orange points represent individual measures of cells exposed to triclosan as a control, with n = 12 at concentration 0µg/µl and n = 6 for all other concentrations. Letters represent statistical categories from a tukey HSD posthoc test after ANOVA (**Supplementary File 2**). Boxplots show means with 25th and 75th percentile boxes, and minimum and maximum values as whiskers, with outliers represented by black points.

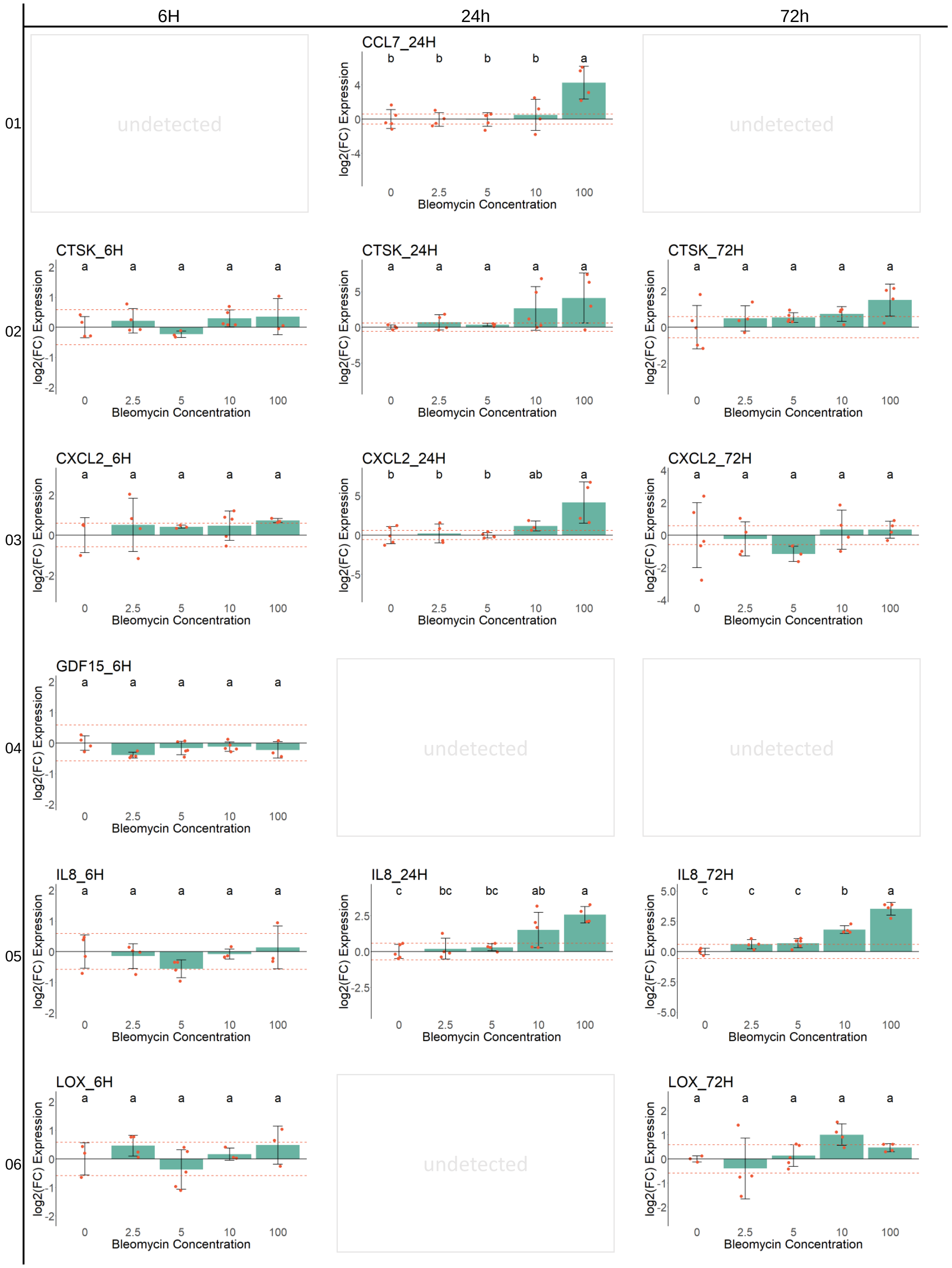
6H
24h
72h
01
02
03
04
05
06
undetected
CCL7_24H
b b b b a
undetected
CTSK_6H
a a a a a
CTSK_24H
a a a a a
CTSK_72H
a a a a a
CXCL2_6H
a a a a a
CXCL2_24H
b b b ab a
CXCL2_72H
a a a a a
GDF15_6H
a a a a a
undetected
undetected
IL8_6H
a a a a a
IL8_24H
c bc bc ab a
IL8_72H
c c c b a
LOX_6H
a a a a a
undetected
LOX_72H
a a a a a
log2(FC) Expression
0 2.5 5 10 100
Bleomycin Concentration

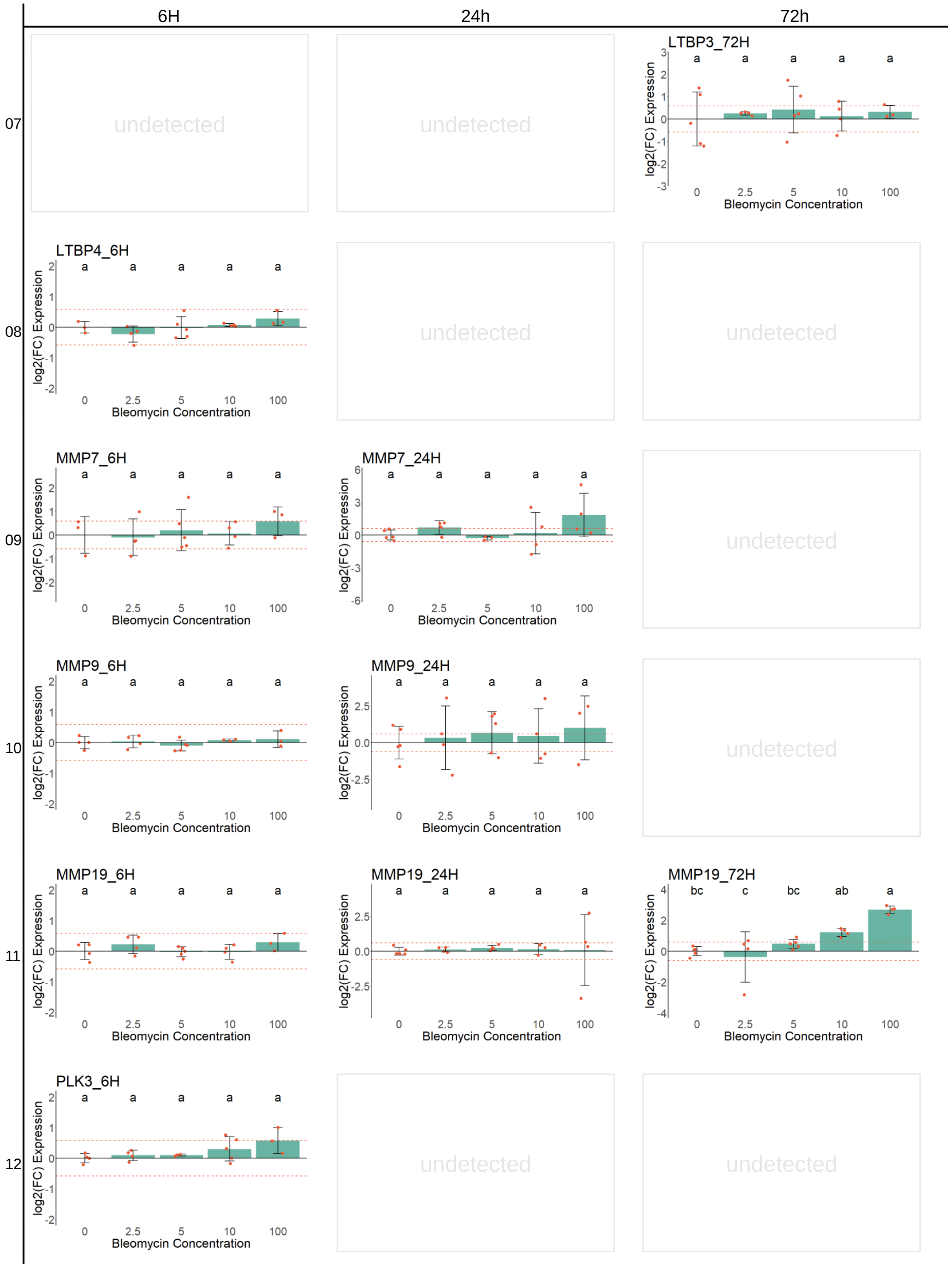
6H
24h
72h
07
08
09
10
11
12
undetected
LTBP3_72H
LTBP4_6H
MMP7_6H
MMP7_24H
MMP9_6H
MMP9_24H
MMP19_6H
MMP19_24H
MMP19_72H
PLK3_6H
a
bc
c
ab
log2(FC) Expression
Bleomycin Concentration
0
2.5
5
10
100

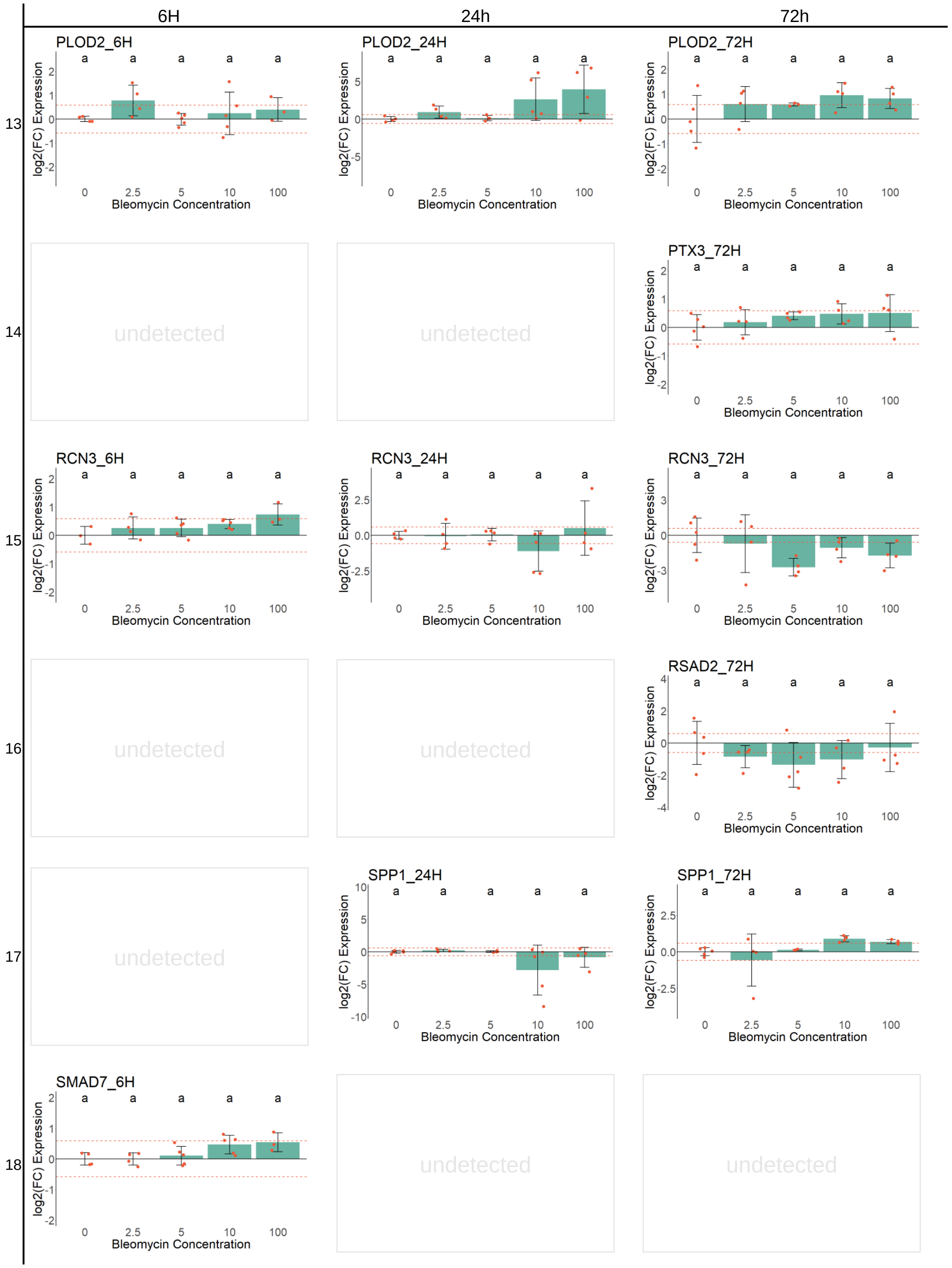
6H
24h
72h
13
PLOD2_6H
PLOD2_24H
PLOD2_72H
14
undetected
undetected
PTX3_72H
15
RCN3_6H
RCN3_24H
RCN3_72H
16
undetected
undetected
RSAD2_72H
17
undetected
SPP1_24H
SPP1_72H
18
SMAD7_6H
undetected
undetected
log2(FC) Expression
Bleomycin Concentration
0
2.5
5
10
100
a

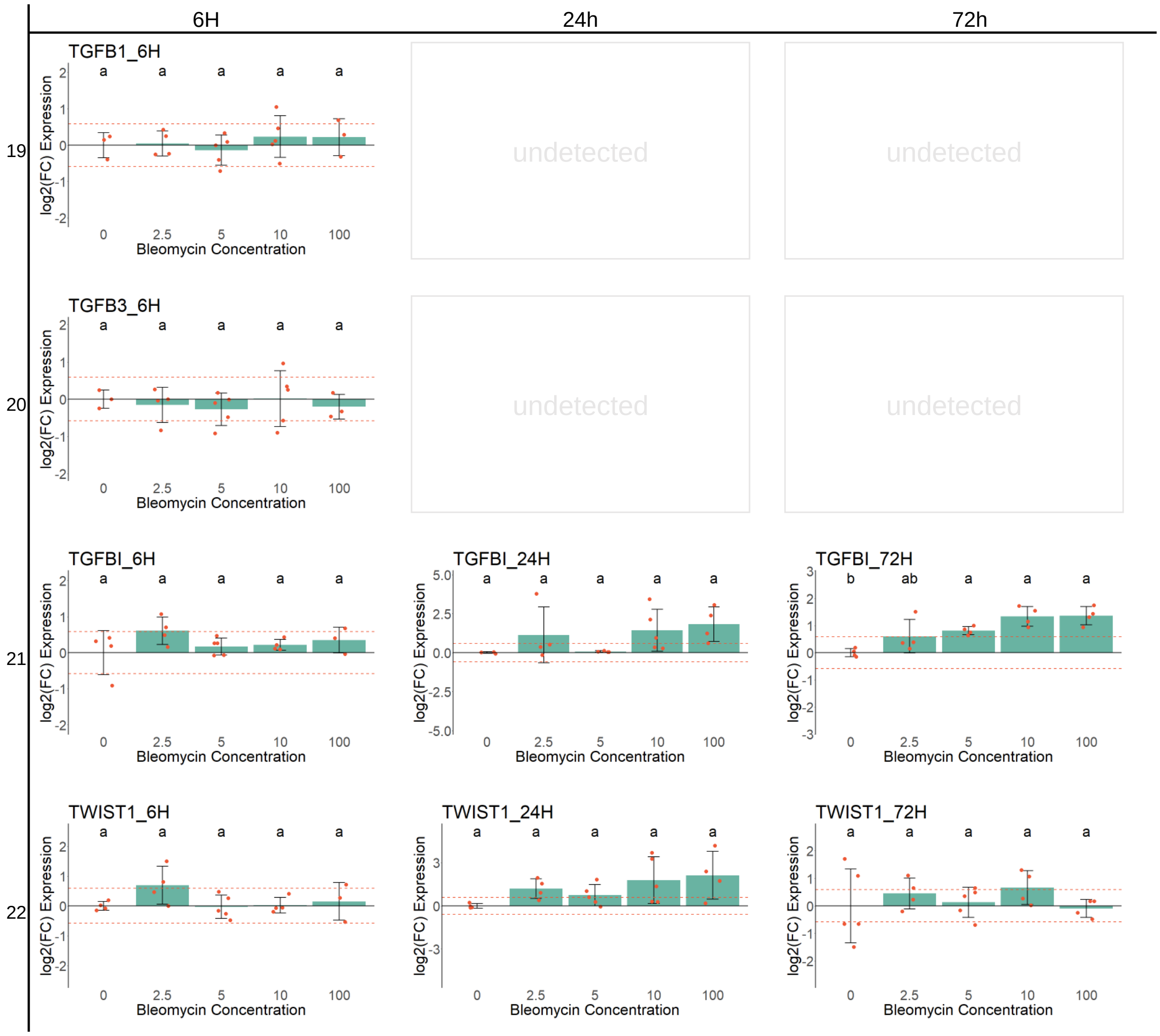

Figure S4. mRNA expression of candidate biomarkers in THP-1 cells exposed to bleomycin for 6, 24 and 72hrs. Each gene plotted as mean log2 fold change (FC) values, ± SD, at 5 concentrations (µg/ml) of bleomycin exposure, relative to ACTB housekeeping gene expression, normalized to bleomycin concentration 0µg/ml (set to mean 0 log2(FC)). Orange points represent individual measures with an n range of 3-5. The orange dashed lines represent a fold change of 1.5 and -1.5 (log2(1.5) and –log2(1.5) respectively). Letters represent statistical categories from a tukey HSD posthoc test after ANOVA (**Supplementary File 2**).

Supplementary Files

Supplementary File 1. Excel file containing reference chemicals with ther CAS numbers and groupings on the first sheet. Following sheets contain the top five enriched AOPs ordered by adjusted p-value. Order of the sheets follow the order of the table on the first sheet.

Supplementary File 2. Excel file reporting the results of the WST and RT-qPCR experiments together with the statistical analyses applied for the data on separate sheets.

Supplementary File 3. Excel file reporting the global rank of the pulmonary fibrosis (PF) genes with the experimental data used for biomarker selection on the first sheet, and the ranked gene lists for individual sheets for each PF KE on the following sheets.
